# Description of combined ARHSP/JALS phenotype in some patients with *SPG11* mutations

**DOI:** 10.1002/mgg3.1240

**Published:** 2020-05-08

**Authors:** Marzieh Khani, Hosein Shamshiri, Farzad Fatehi, Mohammad Rohani, Bahram Haghi Ashtiani, Fahimeh Haji Akhoundi, Afagh Alavi, Hamidreza Moazzeni, Hanieh Taheri, Mina Tolou Ghani, Leila Javanparast, Seyyed Saleh Hashemi, Ramona Haji‐Seyed‐Javadi, Matineh Heidari, Shahriar Nafissi, Elahe Elahi

**Affiliations:** ^1^ School of Biology College of Science University of Tehran Tehran Iran; ^2^ Department of Neurology Shariati Hospital, Tehran University of Medical Sciences Tehran Iran; ^3^ Department of Neurology Hazrat Rasool Hospital Iran University of Medical Sciences Tehran Iran; ^4^ Department of Neurology Firoozgar Hospital Iran University of Medical Sciences Tehran Iran; ^5^ Genetics Research Center University of Social Welfare and Rehabilitation Sciences Tehran Iran; ^6^ Department of Radiation Oncology Emory University School of Medicine Atlanta GA USA

**Keywords:** ALS, ARHSP, autosomal recessive hereditary spastic paraplegia, juvenile amyotrophic lateral sclerosis, *SPG11*

## Abstract

**Background:**

*SPG11* mutations can cause autosomal recessive hereditary spastic paraplegia (ARHSP) and juvenile amyotrophic lateral sclerosis (JALS). Because these diseases share some clinical presentations and both can be caused by *SPG11* mutations, it was considered that definitive diagnosis may not be straight forward.

**Methods:**

The DNAs of referred ARHSP and JALS patients were exome sequenced. Clinical data of patients with *SPG11* mutations were gathered by interviews and neurological examinations including electrodiagnosis (EDX) and magnetic resonance imaging (MRI).

**Results:**

Eight probands with *SPG11* mutations were identified. Two mutations are novel. Among seven Iranian probands, six carried the p.Glu1026Argfs*4‐causing mutation. All eight patients had features known to be present in both ARHSP and JALS. Additionally and surprisingly, presence of both thin corpus callosum (TCC) on MRI and motor neuronopathy were also observed in seven patients. These presentations are, respectively, key suggestive features of ARHSP and JALS.

**Conclusion:**

We suggest that rather than ARHSP or JALS, combined ARHSP/JALS is the appropriate description of seven patients studied. Criteria for ARHSP, JALS, and combined ARHSP/JALS designations among patients with *SPG11* mutations are suggested. The importance of performing both EDX and MRI is emphasized. Initial screening for p.Glu1026Argfs*4 may facilitate *SPG11* screenings in Iranian patients.

## Graphical abstract

Nineteen patients of eight families with *SPG11* mutations are described. Two previously unreported *SPG11* mutations are reported. Definitive diagnosis of ARHSP or JALS is sometimes very difficult. Rather than ARHSP or JALS, combined ARHSP/JALS is the appropriate description for some patients with *SPG11* mutation. Criteria for ARHSP, JALS, and combined ARHSP/JALS designations among patients with *SPG11* mutations are suggested.

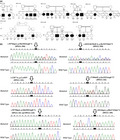

## 1 INTRODUCTION

Mutations in *SPG11* (OMIM 610844) which encodes spatacsin (after “spasticity with thin or atrophied corpus callosum syndrome protein”) are associated with various neurological diseases. The gene was first identified as a major cause of autosomal recessive hereditary spastic paraplegia (ARHSP; OMIM 604360) in 2007(Stevanin et al., 2007). It was shown that *SPG11* is expressed ubiquitously in the nervous system and most prominently in the cerebellum, cerebral cortex, hippocampus, and pineal gland. Very quickly after the initial publication, *SPG11* mutations were reported in ARHSP patients of various ethnicities (Boukhris et al., 2008; Del Bo et al., 2007; Denora et al., 2009; Hehr et al., 2007; Lee et al., 2008; Paisan‐Ruiz, Dogu, Yilmaz, Houlden, & Singleton, 2008; Zhang et al., 2008). Hereditary spastic paraplegia (HSP) constitutes a clinically and genetically heterogeneous group of disorders whose clinical hallmark is progressive spasticity and weakness, which are more prominent in the lower limbs (Novarino et al., 2014; Schule & Schols, 2011). Additional indications include increased tendon reflexes, bilateral Babinski sign, muscle weakness, and urinary urgency. HSP is classically associated with only upper motor neuron (UMN) defects. Its prevalence is estimated to be 2‐10/100000 in most populations (Ruano, Melo, Silva, & Coutinho, 2014). The disease is classified as pure when spasticity and weakness occur in isolation or as complex when the spasticity and weakness are accompanied by other impairments including thinning of the corpus callosum (TCC), cognitive impairment, amyotrophy, ataxia, peripheral neuropathy, pseudobulbar involvement, deafness, and retinal manifestations (Harding, 1983; Stevanin et al., 2008). Clearly, complex HSP as compared to pure HSP is a more severe disorder. Although inheritance of HSP in familial forms can be autosomal dominant (AD), autosomal recessive (AR), or X‐linked, inheritance of pure HSP is usually AD and inheritance of complex HSP is usually AR. At least 58 ARHSP‐causing genes have been identified, but *SPG11* mutations were reported as a common cause in various studies and sometimes accounted for disease in approximately 50% of the patients investigated (Dong et al., 2018; Du, Hu, Tang, Jiang, & Shen, 2018; Kara et al., 2016; Schule et al., 2016).

Adult onset amyotrophic lateral sclerosis (ALS; OMIM 105400) is a progressive and fatal motor neuron disease with an estimated prevalence of 2‐7/100000 (Nelson, 1995; Wijesekera & Leigh, 2009). Death usually ensues 3–5 years after onset of symptoms. ALS is characterized by dysfunction and degeneration of both UMNs in the cortex and lower motor neurons (LMNs) in the brainstem and spinal cord. Like HSP, initial manifestations of motor neuron symptoms usually involve the limbs. UMN damage causes spasticity in the arms and legs that lead to difficulty in writing, walking, uncoordinated movements, and brisk reflexes. LMN damage causes weakness, muscle wasting, and fasciculation. Approximately 5%‐10% of cases are familial, and inheritance pattern in most of these is AD (Dion, Daoud, & Rouleau, 2009). Juvenile onset ALS (JALS) is a rare form of ALS with onset before the age of 25 years and an AR pattern of inheritance. In addition to recessive inheritance and early onset, JALS is typically distinguished from adult onset ALS by its slow rate of progression and long disease duration of up to a few decades (Hentati et al., 1994). Overlaps between clinical features of ARHSP and JALS are evident (Fink, 2001; Meyer et al., 2005; Strong & Gordon, 2005). In addition to shared clinical presentations, pathological findings and genetic evidence also support shared etiologies for the two diseases. For example, mutations in the ARHSP gene *ERLIN2* (SPG18) have been reported in JALS patients and mutations in the JALS gene *Alsin* (ALS2) have been reported as the cause of ARHSP (Al‐Saif, Bohlega, & Al‐Mohanna, 2012; Eymard‐Pierre et al., 2002; Hadano et al., 2001; Wakil et al., 2014; Yang et al., 2001; Yildirim et al., 2011). Yet, results of electrodiagnostic (EDX) testing are usually not the same for ARHSP‐diagnosed patients and JALS‐diagnosed patients. Specifically, motor neuronopathy is always evidenced by electromyography (EMG) in JALS patients. Nerve conduction studies (NCS) are more likely to suggest abnormal sensory findings in ARHSP patients as compared to JALS patients. Nevertheless, similarities between clinical features of ARHSP and JALS prompted mutation screening of *SPG11* in 25 families with JALS‐affected members; patients of 10 families were found to have mutations in *SPG11* (Orlacchio et al., 2010). Mutations in *SPG11* were subsequently identified by exome sequencing in two additional JALS‐affected families without mutations in known ALS‐causing genes (Daoud et al., 2012).

Charcot–Marie–Tooth (CMT) disease constitutes a heterogeneous group of inherited peripheral neuropathies (Mathis et al., 2015) with an estimated prevalence of one in a few thousand in most populations (Gonzaga‐Jauregui et al., 2015). The clinical features of adult‐onset CMT are highly variable but usually include symmetric slowly progressive distal muscle weakness and atrophy that first affect the lower limbs, foot deformities, slight or moderate distal sensory impairment, and depressed tendon reflexes (Lupski et al., 2010; Marttila et al., 2017; Mathis et al., 2015). CMT is commonly classified as CMT type 1 (CMT1; demyelinating) or CMT type 2 (CMT2; axonal). As compared to JALS/ARHSP, overlap of clinical features between CMT and ARHSP and also between CMT and JALS is less. Spasticity and increased deep tendon reflexes, which are among the presentations associated with both ARHSP and JALS, are not among the clinical features of CMT. In fact, CMT is characterized by diminished tendon reflexes. Nevertheless, the presence of TCC in three siblings affected with AR axonal CMT (ARCMT2) prompted *SPG11* mutation screening in these siblings and in 27 other ARCMT2 families without mutations in other candidate genes (Montecchiani et al., 2016). *SPG11* mutations were reported in 12 of the families. Finally, Parkinsonism, cerebellar ataxia, and even multiple sclerosis‐like symptoms have been reported in some patients with *SPG11* mutations, thus adding to the clinical heterogeneity that is associated with mutations in *SPG11* (Balicza et al., 2018; Faber et al., 2018; Guidubaldi et al., 2011; Mukai et al., 2018).

*SPG11* is a large gene that has 40 exons and spans approximately 100 kb; it encodes a protein of 2,443 amino acids. Although the function of spatacsin with respect to disease etiology is not well known, it has been suggested to have roles in axonal maintenance and trafficking, and also in recycling of lysosomes from autolysosomes (Chang, Lee, & Blackstone, 2014; Hirst et al., 2015; Perez‐Branguli et al., 2014). Over 250 mutations in the gene have been reported, the vast majority of which are nonsense, splicing, or frameshift mutations. These are likely loss of function mutations as they cause diseases with recessive inheritance and as the mutated alleles are expected to encode truncated proteins. They are distributed throughout the length of the gene; there are no obvious hot spots of mutation, and there are no regions whose mutations are associated specifically with ARHSP, JALS, or ARCMT2 (Montecchiani et al., 2016). In fact, several of the reported mutations have been observed in two or even all three of the diseases. It has been suggested that the pattern of observed *SPG11* mutations may in part be due to the presence of multiple Alu sequences in *SPG11* that could promote nonallelic homologous recombination and mutational events during replication (Baskin, Kalia, Banwell, Ray, & Yoon, 2017). The findings summarized above suggest partly shared etiologies among the disorders described and beg the question of why individuals bearing *SPG11* mutations have different clinical presentations.

Here, we report finding *SPG11* mutations in 19 available individuals of eight families who are affected with neurological disorders. Subjective, clinical, EDX, and muscle magnetic resonance imaging (MRI) data on the patients are presented. Features common to ARHSP and JALS were present in the patients of all the families. We conclude that diagnosis of ARHSP is in order for patients of one of the families, but that definitive diagnosis of either ARHSP or JALS in the remaining patients is not straightforward. They have both ARHSP and JALS presentations that may somewhat favor one or the other of the two diseases in various patients.

## 2 SUBJECTS AND METHODS

This research was performed in accordance with the Declaration of Helsinki and with approval of the ethics board of the University of Tehran.

HSP‐ or ALS‐diagnosed individuals were referred to us by various neurologists for genetic analysis in the framework of our ongoing studies on ALS in Iranians and our newly initiated studies on HSP (Alavi et al., 2013, 2014; Khani et al., 2019). Genetic analysis was initiated on some of the patients by whole‐exome sequencing of their DNA using the Sure Select V6‐POST kit and an Illumina HiSeq 4,000 system (Illumina, CA, USA). The selected patients were among those who had at least one affected sibling, and whose pedigree structure was consistent with AR inheritance of disease. The sequencing of two probands (SPG11‐300‐II1 and SPG11‐303‐ II1) had been done commercially, and the mutation reports were placed at our disposal. Exome sequence alignment for the other probands was performed by us against human reference genome GRCh37/hg19, and variant callings were done by using ENSEMBL Variant Effect Predictor (http://www.ensembl.org/Tools/VEP) and wANNOVAR (http://wannovar.wglab.org/). Subsequently, filtering was performed by removing SNPs with a minor allele frequency (MAF) of > 0.01 in the dbSNP database (http://www.ncbi.nlm.nih.gov/), the Trans‐Omics for Precision Medicine program (https://www.nhlbiwgs.org/), the 1,000 Genomes database (www.1000genomes.org), the NHLBI Exome Sequencing Project (http://evs.gs.washington.edu/EVS/), the Exome Aggregation Consortium database (http://exac.broadinstitute.org/), the Genome Aggregation Database (http://genomad.broadinstitute.org/), the Greater Middle East Variome Project (http://igm.ucsd.edu/gme/), ENSEMBL (https://www.ensembl.org/index.html), the Healthy Exomes database (https://www.alzforum.org/exomes/hex), the Sequencing Initiative Suomi database (http://www.sisuproject.fi/), the VarCards database (http://varcards.biols.ac.cn/), or the Iranome database (http://iranome.com/), or observed in in‐house exome data belonging to approximately 50 unrelated Iranians affected with non‐neurological diseases. Among the variations that remained, those that did not affect amino acid change or splicing were also removed. Finally, a file of homozygous variations and a file of compound heterozygous variations were prepared. Variations in the files were scrutinized to identify those within any of the 44 known ALS‐causing or 91 known HSP‐causing genes (Table S1). Candidate disease‐causing variations identified were screened for segregation with disease status by direct sequencing. Novel mutations were also screened in 300 Iranian control individuals by an allele‐specific PCR protocol or sought in the Iranome database that contains exome data on 800 healthy Iranians.

Probands with mutations in *SPG11* and their available affected family members were recruited, interviewed, and examined by at least two collaborating neurologists (HS and either SN, FF, MR, or BH). EDX including NCS and needle EMG was done in upper and lower extremities, truncal regions, and cranial regions according to standard procedures (Dantec Keypoint G4, Natus, CA, USA). Brain magnetic resonance imaging (MRI) was performed using a 1.5‐T system (MAGNETOM Avanto 1.5 Tesla, Siemens). T1‐ and T2‐weighted spin‐echo protocols were performed.

## 3 RESULTS

### 3.1 Genetic analysis

**Figure 1:**
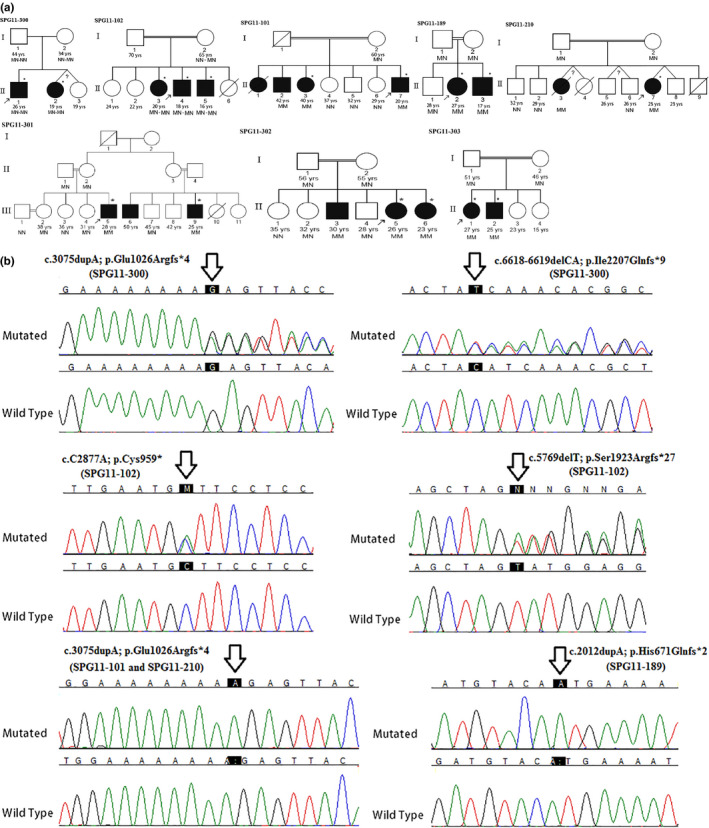
*SPG11* mutations in patients of eight families. (a) Pedigrees of the eight families. *SPG11* genotypes of individuals tested are presented. Individual SPG11‐210‐II3 was genotyped prior to her death. Present age of individuals is provided when known. Filled circles and squares, affected; unfilled circles and squares, unaffected at time of examination. Among the affected individuals, only those designated with * were clinically examined, and the others were reported to be affected by family members. M, mutated *SPG11* allele; N, wild‐type *SPG11* allele; MN‐MN, compound heterozygous *SPG11* phenotypes. (b) DNA sequence chromatograms showing various *SPG11* mutations identified in the eight families

Pedigrees of the eight families who were found to harbor mutations in *SPG11* and whose clinical features were carefully evaluated are shown in Figure 1a. Inheritance of disease in all the families was consistent with an AR pattern, as multiple affected offspring were born to unaffected parents. Parents in seven of the families were consanguineous. Family SPG11‐102 is originally from Afghanistan, and the others are Iranian. Compound heterozygous mutations c.3075dupA (p.Glu1026Argfs*4) and c.6618‐6619delCA (p.Ile2207Glnfs*9) in *SPG11* were reported in the exome sequence results of the proband of family SPG11‐300, and homozygous c.3075dupA (p.Glu1026Argfs*4) mutations were reported for the proband of family SPG11‐303. The filtering protocol applied by us to the whole‐exome sequence data of the probands of six families identified homozygous *SPG11* mutations in five, SPG11‐101, SPG11‐189, SPG11‐210, SPG11‐301, and SPG11‐302 (Figure 1b; Table 1). The specifications of exome data of all patients reflect high quality sequencing (Table S2); the data pertaining to SPG11‐210‐II7 are presented in Table S2. Compound heterozygous mutations in *SPG11* were found in the exome sequence data of the proband of the Afghani family SPG11‐102. Observation of compound heterozygous mutations in SPG11‐102‐II4 was unexpected because the parents of this family reported consanguinity. Neither homozygous nor compound heterozygous mutations in other candidate genes were observed in the exome sequences of the six families (SPG11‐102, SPG11‐101, SPG11‐189, SPG11‐210, SPG11‐301, and SPG11‐302) that we ourselves had sequenced and were also not reported in the results of SPG11‐300 and SPG11‐303 (Table S1). All the *SPG11* mutations segregated with disease status in the respective families (Figure 1a). Interestingly, the same p.Glu1026Argfs*4‐causing mutation (c.3075dupA) was observed in the homozygous state in families SPG11‐101, SPG11‐210, SPG11‐301, SPG11‐302, and SPG11‐303. It was also observed as one of two mutated alleles in family SPG11‐300. This mutation has previously been reported in several studies (Balicza et al., 2018; Denora et al., 2009; Hehr et al., 2007; Orlacchio et al., 2010; Orlen et al., 2009; Schneider‐Gold et al., 2017), usually in association with ARHSP diagnosis and once in a JALS diagnosis (Orlacchio et al., 2010). Three different intragenic haplotypes are associated with the p.Glu1026Argfs*4‐causing mutation among the families that carry it in the homozygous state. This suggests that among the families studied, there are three different *SPG11* alleles with the c.3075dupA mutation that are not identical by descent (Table 2). Unfortunately, inclusion of the p.Glu1026Argfs*4‐causing mutated alleles of families SPG11‐300 and SPG11‐303 in the haplotype analysis was not possible because the full exome data of the probands of these families were not available to us. The c.3075dupA nucleotide appears to be a mutation hotspot; it is positioned within a run of eight A nucleotides in the wild‐type allele, and such runs promote slippage events during replication that can result in deletions and additions. In any case, presence of the p.Glu1026Argfs*4‐causing mutated allele in six of the seven Iranian families here studied suggests that this may be a common *SPG11* mutation among Iranian patients. The two *SPG11* mutations found in family SPG11‐102 were each infrequently previously reported in the homozygous state, and always in HSP‐diagnosed patients (https://www.ncbi.nlm.nih.gov/; Paisan‐Ruiz et al., 2008; Stevanin et al., 2008). The p.Ile2207Glnfs*9‐ and p.His671Glnfs*2‐causing mutations, respectively, of families SPG11‐300 and SPG11‐189 have not been previously reported. They were absent in the chromosomes of 300 Iranian control individuals and in the Iranome database. The American College of Medical Genetics (ACMG) classified all five observed *SPG11* mutations including one nonsense and four frameshift mutations as pathogenic (http://wintervar.wglab.org/mlr.php).

**Table 1:** Phenotypic features and *SPG11* genotypes of patients from eight families with *SPG11* mutations (patients available were examined)

Family	SPG11‐300		SPG11‐102			SPG11‐101		SPG11‐189		SPG11‐210
Origin of family	Orumieh, Iran		Afghanistan			Zanjan, Iran		Ghazvin, Iran		Kurdistan, Iran
	SPG11‐300‐II1	SPG11‐300‐II2	SPG11‐102‐II3	SPG11‐102‐II4	SPG11‐102‐II5	SPG11‐101‐II3	SPG11‐101‐II7	SPG11‐189‐II2	SPG11‐189‐II3	SPG11‐210‐II7
Age at examination (years)	26	19	20	18	16	40	20	27	17	23
Age at onset (years)	16	17	12	12	13	22	16	17	12	15
Disease duration (years)^a^	10	2	8	6	3	18	4	10	5	8
Sex	Male	Female	Female	Male	Male	Female	Male	Female	Male	Female
Initial manifestation	Leg weakness & stifness	Leg weakness & stifness	Leg weakness & stifness	Hand tremor	Leg weakness & stifness	Leg weakness & stifness	Leg weakness & stifness	Leg weakness & stifness	Leg weakness & stifness	Leg weakness & stifness
Motor disturbance	Weakness^c^	Weakness^c^	Weakness^b^	Weakness^b^	Weakness^b^	Weakness^b^	Weakness^b^	Weakness^b^	Weakness^c^	Weakness^c^
Spasticity	+	+	+	+	+	+	+	+	+	+
Distal amyotrophy	+	−	+	+	+	+	+	+	+	+
Dysarthria	−	−	−	+ (mild)	+ (mild)	+ (mild)	−	−	−	−
Dysphagia	−	−	−	−	−	−	−	−	−	−
Subjective sensory symptoms	−	−	−	−	−	−	−	−	−	−
Sensory signs	Normal exam	Normal exam	+^d^	+^d^	+^d^	Normal exam	Normal exam	Normal exam	Normal exam	Normal exam
Deep tendon reflexes	Increased	Increased	Increased	Increased	Increased	Increased	Increased	Increased	Increased	Increased
Tremor	−	−	−	+	+	−	−	−	−	−
Urinary incontinence	−	−	−	−	−	−	−	−	−	−
Ataxia	−	−	−	−	−	−	−	−	−	−
Mental impairment	−	−	+	+	+	−	−	−	−	−
Ambulatory state	Needs walking device	independent	Needs help	Needs help	Needs walking device	Needs walking device	Slow but independent	Needs walking device	Slow but independent	Bedridden
EMG	Normal	Normal	Motor neuronopathy^e^	Motor neuronopathy^e^	Motor neuronopathy^e^	Motor neuronopathy^e^	Motor neuronopathy^e^	Motor neuronopathy^e^	Motor neuronopathy^e^	Motor neuronopathy^e^
NCS	Normal sensory	Normal sensory findings	Mild sensory polyneuropathy^1^	Mild sensory polyneuropathy^2^	Mild sensory polyneuropathy^2^	Mild sensory polyneuropathy^3^	Normal sensory findings	Normal sensory findings	Normal sensory findings	Normal sensory findings
Brain MRI	TCC & T2 periventricular hyperintensity^f^	TCC & T2 periventricular hyperintensity^f^	TCC & T2 periventricular hyperintensity^f^	TCC & T2 periventricular hyperintensity^f^	TCC & T2 periventricular hyperintensity^f^	TCC & T2 periventricular hyperintensity^f^	TCC & T2 periventricular hyperintensity^f^	TCC & T2 periventricular hyperintensity^f^	TCC & T2 periventricular hyperintensity^f^	TCC & T2 periventricular hyperintensity^f^
*SPG11* mutations	Compound heterozygous		Compound heterozygous			Homozygous		Homozygous		Homozygous
	c.3075dupA; p.Glu1026Argfs*4		c.C2877A; p.Cys959*			c.3075dupA; p.Glu1026Argfs*4		c.2012dupA; p.His671Glnfs*2		c.3075dupA;
	c.6618‐6619delCA; p.Ile2207Glnfs*9		c.5769delT; p.Ser1923Argfs*27							p.Glu1026Argfs*4

Family	SPG11‐301		SPG11‐302		SPG11‐303					
Origin of family	Kermanshah, Iran		Zanjan, Iran		Tabriz, Iran					
	SPG11‐301‐III5	SPG11‐301‐III9	SPG11‐302‐II5	SPG11‐302‐II6	SPG11‐303‐II1	SPG11‐303‐II2				
Age at examination (years)	22	30	26	23	27	25				
Age at onset (years)	15	5	15	15	18	20				
Disease duration (years)^a^	7	25	11	8	9	5				
Sex	Male	Male	Female	Female	Female	Male				
Initial manifestation	Leg weakness & stifness	Leg weakness & stifness	Leg weakness & stifness	Leg weakness & stifness	Leg weakness & stifness	Leg weakness & stifness				
Motor disturbance	Weakness^c^	Weakness^c^	Weakness^c^	Weakness^c^	Weakness^c^	Weakness^c^				
Spasticity	+	+	+	+	+	+				
Distal amyotrophy	+	+	+	+	+	+				
Dysarthria	+	+	+	−	+	+				
Dysphagia	−	−	−	−	−	−				
Subjective sensory symptoms	−	−	−	−	−	−				
Sensory signs	Normal exam	Normal exam	Normal exam	Normal exam	Normal exam	Normal exam				
Deep tendon reflexes	Increased	Increased	Increased	Increased	Increased	Increased				
Tremor	+	−	−	−	−	−				
Urinary incontinence	−	−	+	−	−	−				
Ataxia	−	−	−	−	−	−				
Mental impairment	−	−	−	−	−	−				
Ambulatory state	Needs walking device	Wheel chair bound	Slow but independent	Slow but independent	Needs help	Slow but independent				
EMG	Motor neuronopathy^e^	Motor neuronopathy^e^	Motor neuronopathy^e^	Motor neuronopathy^e^	Motor neuronopathy^e^	Motor neuronopathy^e^				
NCS	Normal sensory findings	Normal sensory findings	Normal sensory findings	Normal sensory findings	Normal sensory findings	Normal sensory findings				
Brain MRI	TCC & T2 periventricular hyperintensity^f^	TCC & T2 periventricular hyperintensity^f^	TCC & T2 periventricular hyperintensity^f^	TCC & T2 periventricular hyperintensity^f^	TCC & T2 periventricular hyperintensity^f^	TCC & T2 periventricular hyperintensity^f^				
*SPG11* mutations	Homozygous	Homozygous	Homozygous	Homozygous	Homozygous	Homozygous				
	c.3075dupA;	c.3075dupA;	c.3075dupA;	c.3075dupA;	c.3075dupA;	c.3075dupA;				
	p.Glu1026Argfs*4	p.Glu1026Argfs*4	p.Glu1026Argfs*4	p.Glu1026Argfs*4	p.Glu1026Argfs*4	p.Glu1026Argfs*4				

Abbreviations: EMG, electromyography; MRI, magnetic resonance imaging; TCC, thin corpus callosum.

^a^ Yrs between onset and examination.

^b^ More prominent in lower limbs.

^c^ Weakness in lower limbs.

^d^ Decreased vibration sense in lower extremities.

^e^ At extremities and cranial and truncal levels.

^f^ More prominent in frontal region.

^1^ SNAP in upper limbs at lower normal limit and unobtainable SNAPs in lower extremities.

^2^ Decreased SNAP amplitude in upper limbs and unobtainable SNAPs in lower extremities.

^3^ Decreased SNAP in upper & lower limbs. NCS, nerve conduction studies.

**Table 2:** Proposed haplotypes associated with of *SPG11* mutation c.3075dupA (p.Glu1026Argfs*4) in four Iranian families

rs# of variation	Chromosome 15 position (hg 19/GRCh37.p13)	Position	MAF^a^	Effect on protein	Genotype in SPG11‐101	Genotype in SPG11‐210 & SPG11‐301	Genotype in SPG11‐302	Proposed haplotype in SPG11‐101	Proposed haplotype in SPG11‐210 & SPG11‐301	Proposed haplotype in SPG11‐302
rs8040992	44832193	c.147+2568C>A	C=0.0236	—	A/A	C/C	C/C	A	C	C
Novel	44842313	c.148‐762G>T	—	—	T/T	G/G	G/G	T	G	G
rs2556565	44844262	c.294+542A>G	A=0.1354	—	G/G	A/A	A/A	G	A	A
rs312262752^b^	44905697	c.3075dupA	—	p.Glu1026Argfs*4	A/A	A/A	A/A	A	A	A
Novel	44913167	c.2244+373C>A	—	—	A/A	C/C	C/C	A	C	C
rs2412911	44943094	c.1456+595C>T	T=0.2821	—	T/T	C/C	T/T	T	C	T
rs12901431	44962599	c.658‐315A>G	A=0.0787	—	G/G	A/A	G/G	G	A	G
rs10714081	44965946	c.304‐372delA	A=0.0094	—	delA/delA	delA/delA	delA/delA	delA	delA	delA
rs8026845	44966389	c.262A>G	A=0.0096	p.Met88Val	G/G	G/G	G/G	G	G	G
rs2264238	44995211	g.44703013A>G	A=0.0062	—	G/G	A/A	A/A	G	A	A

^a^ Minor allele frequency in dbSNP (https://www.ncbi.nlm.nih.gov/snp).

^b^ Disease causing mutation.

### 3.2 Clinical features of patients with *SPG11* mutations

**Figure 2:**
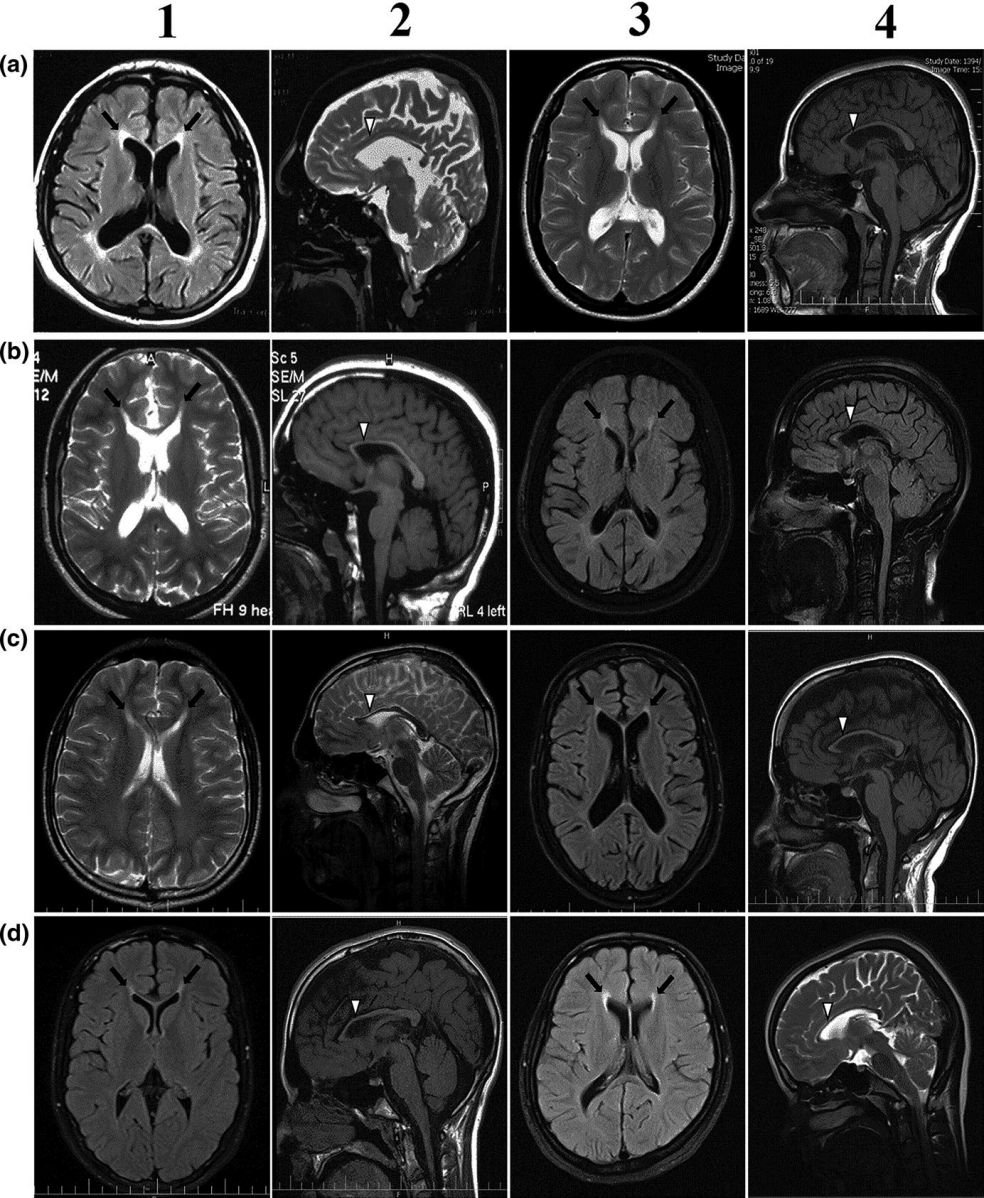
Representative brain MRI images of patients with *SPG11* mutations. a1 and a2, SPG11‐300‐II1; a3 and a4, SPG11‐102‐II3; b1 and b2, SPG11‐102‐II4; b3 and b4, SPG11‐101‐II3; c1 and c2, SPG11‐101‐II7; c3 and c4, SPG11‐189‐II2; d1 and d2, SPG11‐189‐II3; d3 and d4, SPG11‐210‐II7. Rows 1 and 3, axial views; rows 2 and 4, sagittal views. Hyperintensities, which are more prominent in anterior regions next to frontal horns (black arrows), are evident in the axial images. Thin corpus callosum, which is significantly more prominent in anterior/rostral parts (white arrow heads), is shown in the sagittal images

Relevant information on patients of families with *SPG11* mutations is presented in Tables 1 and 3 and in the text below. Representative brain MRI images of patients of the families are shown in Figure 2. The images testify to the interpretation of the images as reported in Table 1.

**Table 3:** ARHSP and AR‐JALS phenotypic features of patients from eight families with *SPG11* mutations^a^

Patient(s)	SPG11‐300‐II1 & SPG11‐300‐II2	SPG11‐102‐II3	SPG11‐102‐II4 & SPG11‐102‐II5	SPG11‐101‐II3	SPG11‐101‐II7	SPG11‐189‐II2 & SPG11‐189‐II3	SPG11‐210‐II7	SPG11‐301‐III5	SPG11‐301‐III9	SPG11‐302‐II5 & SPG11‐302‐II6	SPG11‐303‐II1 & SPG11‐303‐II2
**Findings more suggestive of SPG11 associated ARHSP**											
TCC and T2 periventricular hyperintencities in brain MRI	+	+	+	+	+	+	+	+	+	+	+
Absence of neuronopathy in EDX	+										
Abnormal sensory findings in NCS		+ (mild)	+ (mild)	+ (mild)							
Abnormal sensory examination		+	+								
Mental impairment		+	+								
Tremor		+						+			
**Findings more suggestive of SPG11 associated AR‐JALS**											
EMG evidence of motor neuronopathy		+	+	+	+	+	+	+	+	+	+
Normal sensory findings in NCS	+				+	+	+	+	+	+	+
Normal sensory examination	+			+	+	+	+	+	+	+	+
Normal mental status	+			+	+	+	+	+	+	+	+
**Findings common to SPG11 associated ARHSP & AR‐JALS**											
Motor disturbance	+	+	+	+	+	+	+	+	+	+	+
Spasticity	+	+	+	+	+	+	+	+	+	+	+
Increased deep tendon reflexes	+	+	+	+	+	+	+	+	+	+	+
Long duration^b^	+	+	+	+	+	+	+	+	+	+	+
Presently proposed diagnosis	ARHSP	Combined ARHSP/JALS									
Diagnosis by referring neurologist	HSP	HSP	HSP	JALS	HSP	JALS	JALS	HSP	HSP	HSP	JALS

Abbreviations: EMG, electromyography; MRI, magnetic resonance imaging; NCS, nerve conduction studies; TCC, thin corpus callosum.

^a^ Presentations present indicated with + sign.

^b^ At least one patient in family has disease duration ≥ 8 years.

#### 3.2.1 Family SPG11‐300

The proband (SPG11‐300‐II1) was referred at the age of 26 years with presentations of difficulties in walking and spasticity in lower extremities. Onset of symptoms was at age of 16 years. His mental status was normal, there were no signs of bulbar involvement, sensory signs were normal, and amyotrophy was not seen. EDX evaluation was normal and there was no evidence of lower motor neuronopathy or of sensory neuropathy. Absence of motor neuronopathy in the EDX findings does not support ALS diagnosis. Brain MRI demonstrated TCC, more prominent in the rostral (anterior) region, and periventricular white matter hyperintensities. The proband had an affected 19 years old sister with similar history and presentations since the age of 17 years. We consider that the siblings have pure UMN involvement and are affected with ARHSP.

#### 3.2.2 Family SPG11‐102

The proband (SPG11‐102‐II4) had difficulty in grasping and holding onto objects, and also in performing fine tasks with his hands from age of 12 years. Stiffness and then weakness in lower limbs ensued within 1 year. His speech is low tone, but comprehensible. He does not have swallowing, hearing, or vision problems. Spinal deformity, scoliosis, and dystonic posture in the arms were evident. The patient could not continue schooling after primary school because of mental insufficiency. EDX results indicated prominent motor neuronopathy. The proband has two siblings with similar history and manifestations, except that their mental impairment was more serious, pes cavus was observed, and scoliosis was not evidenced in these individuals. Some phenotypic features that are presented here and in Table 1, particularly the presence of TCC, mental impairment, and some sensory anomaly, argue in favor of ARHSP diagnosis for the SPG11‐102 patients. Yet, EDX evidence of prominent lower motor neuronopathy is suggestive of JALS. The patients apparently have mixed ARHSP and AR‐JALS presentations, slightly more in favor of ARHSP.

#### 3.2.3 Families SPG11‐101, SPG11‐189, SPG11‐210, SPG11‐301, SPG11‐302, and SPG11‐303

Both affected siblings in families SPG11‐189 and SPG11‐303 are described in Tables 1 and 3, whereas all affected siblings of families SPG11‐101, SPG11‐210, SPG11‐301, and SPG11‐302 are not described. SPG11‐101‐II1, who is not described, became symptomatic at age of 18 years and died 27 years later. SPG11‐101‐II2 did not consent to undergo medical examination; onset of symptoms in this patient was at age of 18 years and he is presently 42 years old. SPG11‐210‐II3, who is also not included in the tables, became symptomatic at age of 15 years and died 7 years later. SPG11‐301‐III6 also did not consent to medical examination; onset of symptoms in this patient was at age of 15 years and he is presently 50 years old. SPG11‐302‐II3 could not be brought to Tehran for examination because of complications in transport; he is bedridden and more severely affected than his sisters. The presentations of the 11 examined patients in these six families were very similar. Age of onset of symptoms was in the second or early third decade of life and leg weakness and stiffness were the initial presentations in all the patients. Spasticity, increased deep tendon reflexes, and distal amyotrophy were also present in all the patients. Dysarthria was evidenced in six of the 11 patients. Sensory signs were normal and NCS results showed mild sensory polyneuropathy in only one patient (SPG11‐101‐II3). There were no reports of visual problems in any of the patients. EDX showed motor neuronopathy in all the patients; amplitude of motor action potentials was always in the normal range and EMG showed chronic neurogenic pattern. TCC was present in the brain MRI of all the patients. The clinical features of the patients of these six families did not allow a definitive diagnosis of either ARHSP or JALS (Table 3). Observation of rostral‐predominant TCC in the MRIs is suggestive of ARHSP, whereas the normal mental status and prominent lower motor neuronopathy in EMG findings of all examined patients argue against ARHSP and are supportive of JALS diagnosis for these individuals. The patients have mixed ARHSP and AR‐JALS presentations, perhaps slightly more in favor of JALS. Observation of mild abnormal sensory findings in SPG11‐101‐II3 but not in affected sibling SPG11‐101‐II7 may be the result of significantly longer duration of disease in the former (18 as compared to 4 years).

## 4 DISCUSSION

We have reported *SPG11* mutations in eight families with neurological diseases. Five different mutations were identified, two of which (c.6618‐6619delCA; p.Ile2207Glnfs*9 and c.2012dupA; p.His671Glnfs*2) are novel. The others have previously been reported in ARHSP (c.C2877A; p.Cys959* and c.5769delT; p.Ser1923Argfs*27) diagnosed or ARHSP‐ and JALS‐diagnosed (c.3075dupA; p.Glu1026Argfs*4) patients. Considering repeated observation of the p.Glu1026Argfs*4‐causing mutation in the Iranian patients of this study, it seems reasonable to first check for this mutation in *SPG11* screenings of patients of this population.

Variable presentations among patients diagnosed with ARHSP are well known (Kara et al., 2016). Intrafamilial differences in clinical features among patients with *SPG11* mutations have also been reported in several studies (Daoud et al., 2012; Denora et al., 2009; Iskender et al., 2015; Schneider‐Gold et al., 2017). Partial overlap in clinical presentations of ARHSP and JALS has been well noted (Denora et al., 2016; Fink, 2001; Meyer et al., 2005; Strong & Gordon, 2005). In some studies, both ARHSP‐diagnosed patients and JALS‐diagnosed patients were reported in the same family (Daoud et al., 2012; Iskender et al., 2015). Among the families with *SPG11* mutations reported in this study, we have suggested that diagnosis of ARHSP is appropriate for affected individuals of only one family (SPG11‐300). The presentations of the patients of this family including muscle weakness, stiffness, spasticity, and increased reflexes may all be attributed to UMN malfunctioning. Absence of EDX evidence for motor neuronopathy suggests the absence of LMN involvement. Definitive diagnosis of either ARHSP or AR‐JALS is difficult for the patients of the remaining families. Patients of the latter families have some presentations supportive of ARHSP diagnosis, and other features supportive of JALS diagnosis. A combined ARHSP/JALS phenotype may be a better designation for their condition. We emphasize that proposal of a combined ARHSP/JALS phenotype is related to but goes beyond recognition that pure ARHSP and pure JALS share some presentations including motor disturbance, spasticity, increased deep tendon reflexes, and long disease duration. It emphasizes that some patients simultaneously manifest presentations that are classically associated with ARHSP and also presentations classically associated with JALS. The most evident of these are presentation of TCC that is commonly associated with ARHSP and EDX evidence of motor neuronopathy that is a cardinal feature associated with JALS. Although a relatively small number of families were studied here, it is notable that the condition of the patients in the majority of the families was considered to be the combined ARHSP/JALS phenotype. The importance of correct diagnosis will become more important if effective treatment protocols become available for one or both of the diseases. Presently, riluzole and edaravone are administered only to ALS patients (https://doi.org/10.1016/S1474‐4422(18)30091‐7) (Sawada, 2017). CMT diagnosis was not considered for patients of any of the families studied here, as CMT features, including absence of spasticity and depressed tendon reflexes, were not among the clinical presentations of the patients.

A review of the literature reveals that while recognizing the overlaps in presentations of ARHSP and JALS, only rarely have authors ultimately refrained from making a definitive diagnosis of one or the other in reported patients (Querin et al., 2014). In some cases, justifications of definitive diagnoses based on reported data are not obvious. For example, in one study, patients of three Turkish families with *SPG11* mutations were diagnosed with JALS, and ARHSP was “definitively excluded” in these families (Iskender et al., 2015). However, patients of at least one of the families (Family 3) presented with cognitive impairment and TCC, which are consistent with ARHSP. EDX is not routinely performed in the clinical setting for patients who are readily diagnosed with ARHSP; this precludes detection of EDX evidence of motor neuronopathy if it were to be present. And when EDX is performed, there may be no motivation to specifically query the presence or absence of motor neuronopathy (Kara et al., 2016). Although MRI is often performed for ALS diagnosis, its purpose is usually for differential diagnosis (Sawalha, Gonzalez‐Toledo, & Hussein, 2019). As such, physicians may not carefully focus on the presence or absence of TCC in the images.

Variations in presentations of patients with *SPG11* mutations may be due to environmental factors, stochastic events during development, and of course differences in genetic background pertaining to other genes. In Table 3, the presence of motor disturbances, spasticity, increased deep tendon reflexes, and long disease duration are presented as findings common to both *SPG11*‐associated ARHSP and *SPG11*‐associated AR‐JALS. Within this background, EDX evidence of motor neuronopathy, absence of evidence for sensory anomalies, normal mental status, and absence of TCC in brain MRI are supportive of AR‐JALS diagnosis. Within the shared background, presence of TCC in brain MRI, absence of neuronopathy in EDX, abnormal sensory findings, and mental impairment are supportive of ARHSP diagnosis. In patients with clinical signs of UMN involvement (spasticity, increased DTR, and weakness), EDX evidence of motor neuronopathy is the most important feature that can determine whether LMN involvement is present (as in JALS) or absent (as in ARHSP). This feature becomes evident early in the course of the disease, and is therefore useful for early differential diagnosis. For practical application, we suggest that patients with the shared ARHSP and AR‐JALS presentations described above may be classified as ARHSP if there is no EDX evidence of motor neuronopathy but TCC is evidenced in brain MRI. They may be classified as AR‐JALS if EDX results indicate motor neuronopathy and TCC is not evidenced in brain MRI. Patients with the common ARHSP and AR‐JALS presentations and also EDX evidence of motor neuronopathy and TCC in MRI may be diagnosed with a combined ARHSP/JALS phenotype. In light of the information provided, we suggest that EDX and MRI be performed on patients for whom ARHSP or JALS diagnosis is being considered, and that the presence or absence of neuronopathy and TCC be carefully assessed.

## CONFLICT OF INTEREST

The authors declare that they have no conflict of interest.

## AUTHOR CONTRIBUTION

MK: recruited patients and family members, analysis of exome data, performed segregation analysis in the families by direct Sanger sequencing, contributed to writing of manuscript; HS: significantly contributed to gathering clinical data, contributed to writing of manuscript; FF, MR, BHA, FHA, and SN: identified and introduced patients and provided clinical data; AA, HM, and RHSJ: analysis of exome data; HT, MTG, LJ, and SSH: performed segregation analysis in the families by direct Sanger sequencing; MH: provided clinical data, EE: designed and supervised the research, wrote the manuscript.

## Supporting information

Table S1

Table S2

## References

[mgg31240-bib-0001] Alavi, A. , Nafissi, S. , Rohani, M. , Shahidi, G. , Zamani, B. , Shamshiri, H. , … Elahi, E. (2014). Repeat expansion in C9ORF72 is not a major cause of amyotrophic lateral sclerosis among Iranian patients. Neurobiology of Aging, 35(1), 267.e1–267.e7. 10.1016/j.neurobiolaging.2013.07.016 23962495

[mgg31240-bib-0002] Alavi, A. , Nafissi, S. , Rohani, M. , Zamani, B. , Sedighi, B. , Shamshiri, H. , Elahi, E. (2013). Genetic analysis and SOD1 mutation screening in Iranian amyotrophic lateral sclerosis patients. Neurobiology of Aging, 34(5), 1516.e1–1516.e8. 10.1016/j.neurobiolaging.2012.09.006 23062701

[mgg31240-bib-0003] Al‐Saif, A. , Bohlega, S. , & Al‐Mohanna, F. (2012). Loss of ERLIN2 function leads to juvenile primary lateral sclerosis. Annals of Neurology, 72(4), 510–516. 10.1002/ana.23641 23109145

[mgg31240-bib-0004] Balicza, P. , Grosz, Z. , Bencsik, R. , Illes, A. , Gal, A. , Gezsi, A. , & Molnar, M. J. (2018). Significance of whole exome sequencing in the diagnostics of rare neurological diseases ‐ own experiences through a case presenting with ataxia. Orvosi Hetilap, 159(28), 1163–1169. 10.1556/650.2018.31049 29983107

[mgg31240-bib-0005] Baskin, B. , Kalia, L. V. , Banwell, B. L. , Ray, P. N. , & Yoon, G. (2017). Complex genomic rearrangement in SPG11 due to a DNA replication‐based mechanism. Movement Disorders, 32(12), 1792–1794. 10.1002/mds.27188 29082553

[mgg31240-bib-0006] Boukhris, A. , Stevanin, G. , Feki, I. , Denis, E. , Elleuch, N. , Miladi, M. I. , … Brice, A. (2008). Hereditary spastic paraplegia with mental impairment and thin corpus callosum in Tunisia: SPG11, SPG15, and further genetic heterogeneity. Archives of Neurology, 65(3), 393–402. 10.1001/archneur.65.3.393 18332254

[mgg31240-bib-0007] Chang, J. , Lee, S. , & Blackstone, C. (2014). Spastic paraplegia proteins spastizin and spatacsin mediate autophagic lysosome reformation. Journal of Clinical Investigation, 124(12), 5249–5262. 10.1172/JCI77598 25365221 PMC4348974

[mgg31240-bib-0008] Daoud, H. , Zhou, S. , Noreau, A. , Sabbagh, M. , Belzil, V. , Dionne‐Laporte, A. , … Rouleau, G. A. (2012). Exome sequencing reveals SPG11 mutations causing juvenile ALS. Neurobiology of Aging, 33(4), 839.e5–839.e9. 10.1016/j.neurobiolaging.2011.11.012 22154821

[mgg31240-bib-0009] Del Bo, R. , Di Fonzo, A. , Ghezzi, S. , Locatelli, F. , Stevanin, G. , Costa, A. , … Comi, G. P. (2007). SPG11: A consistent clinical phenotype in a family with homozygous spatacsin truncating mutation. Neurogenetics, 8(4), 301–305. 10.1007/s10048-007-0095-z 17717710

[mgg31240-bib-0010] Denora, P. S. , Schlesinger, D. , Casali, C. , Kok, F. , Tessa, A. , Boukhris, A. , … Santorelli, F. M. (2009). Screening of ARHSP‐TCC patients expands the spectrum of SPG11 mutations and includes a large scale gene deletion. Human Mutation, 30(3), E500–519. 10.1002/humu.20945 19105190

[mgg31240-bib-0011] Denora, P. S. , Smets, K. , Zolfanelli, F. , Ceuterick‐de Groote, C. , Casali, C. , Deconinck, T. , … El Hachimi, K. H. (2016). Motor neuron degeneration in spastic paraplegia 11 mimics amyotrophic lateral sclerosis lesions. Brain, 139(Pt 6), 1723–1734. 10.1093/brain/aww061 27016404 PMC5839621

[mgg31240-bib-0012] Dion, P. A. , Daoud, H. , & Rouleau, G. A. (2009). Genetics of motor neuron disorders: New insights into pathogenic mechanisms. Nature Reviews Genetics, 10(11), 769–782. 10.1038/nrg2680 19823194

[mgg31240-bib-0013] Dong, E.‐L. , Wang, C. , Wu, S. , Lu, Y.‐Q. , Lin, X.‐H. , Su, H.‐Z. , … Lin, X. (2018). Clinical spectrum and genetic landscape for hereditary spastic paraplegias in China. Molecular Neurodegeneration, 13(1), 36 10.1186/s13024-018-0269-1 29980238 PMC6035405

[mgg31240-bib-0014] Du, J. , Hu, Y. C. , Tang, B. S. , Jiang, H. , & Shen, L. (2018). Identification of novel SPG11 mutations in a cohort of Chinese families with hereditary spastic paraplegia. International Journal of Neuroscience, 128(2), 146–150. 10.1080/00207454.2017.1378878 28933964

[mgg31240-bib-0015] Eymard‐Pierre, E. , Lesca, G. , Dollet, S. , Santorelli, F. M. , di Capua, M. , Bertini, E. , & Boespflug‐Tanguy, O. (2002). Infantile‐onset ascending hereditary spastic paralysis is associated with mutations in the alsin gene. American Journal of Human Genetics, 71(3), 518–527. 10.1086/342359 12145748 PMC379189

[mgg31240-bib-0016] Faber, I. , Martinez, A. R. M. , Martins, C. R. Jr , Maia, M. L. , Souza, J. P. , Lourenco, C. M. , … Franca, M. C. Jr (2018). SPG11‐related Parkinsonism: Clinical profile, molecular imaging and l‐dopa response. Movement Disorders, 33(10), 1650–1656. 10.1002/mds.27491 30306626

[mgg31240-bib-0017] Fink, J. K. (2001). Progressive spastic paraparesis: Hereditary spastic paraplegia and its relation to primary and amyotrophic lateral sclerosis. Seminars in Neurology, 21(2), 199–207. 10.1055/s-2001-15265 11442328

[mgg31240-bib-0018] Gonzaga‐Jauregui, C. , Harel, T. , Gambin, T. , Kousi, M. , Griffin, L. B. , Francescatto, L. , … Lupski, J. R. (2015). Exome sequence analysis suggests that genetic burden contributes to phenotypic variability and complex neuropathy. Cell Reports, 12(7), 1169–1183. 10.1016/j.celrep.2015.07.023 26257172 PMC4545408

[mgg31240-bib-0019] Guidubaldi, A. , Piano, C. , Santorelli, F. M. , Silvestri, G. , Petracca, M. , Tessa, A. , & Bentivoglio, A. R. (2011). Novel mutations in SPG11 cause hereditary spastic paraplegia associated with early‐onset levodopa‐responsive Parkinsonism. Movement Disorders, 26(3), 553–556. 10.1002/mds.23552 21381113

[mgg31240-bib-0020] Hadano, S. , Hand, C. K. , Osuga, H. , Yanagisawa, Y. , Otomo, A. , Devon, R. S. , … Ikeda, J.‐E. (2001). A gene encoding a putative GTPase regulator is mutated in familial amyotrophic lateral sclerosis 2. Nature Genetics, 29(2), 166–173. 10.1038/ng1001-166 11586298

[mgg31240-bib-0021] Harding, A. E. (1983). Classification of the hereditary ataxias and paraplegias. Lancet, 321(8334), 1151–1155. 10.1016/s0140-6736(83)92879-9 6133167

[mgg31240-bib-0022] Hehr, U. , Bauer, P. , Winner, B. , Schule, R. , Olmez, A. , Koehler, W. , … Winkler, J. (2007). Long‐term course and mutational spectrum of spatacsin‐linked spastic paraplegia. Annals of Neurology, 62(6), 656–665. 10.1002/ana.21310 18067136

[mgg31240-bib-0023] Hentati, A. , Bejaoui, K. , Pericak‐Vance, M. A. , Hentati, F. , Speer, M. C. , Hung, W.‐Y. , Figlewicz, D. A. , Haines, J. , Rimmler, J. , Ben Hamida, C. , Ben Hamida, M. , Brown, R. H. , & Siddique, T. , …1994). Linkage of recessive familial amyotrophic lateral sclerosis to chromosome 2q33‐q35. Nature Genetics, 7(3), 425–428. 10.1038/ng0794-425 7920663

[mgg31240-bib-0024] Hirst, J. , Edgar, J. R. , Esteves, T. , Darios, F. , Madeo, M. , Chang, J. , … Robinson, M. S. (2015). Loss of AP‐5 results in accumulation of aberrant endolysosomes: Defining a new type of lysosomal storage disease. Human Molecular Genetics, 24(17), 4984–4996. 10.1093/hmg/ddv220 26085577 PMC4527494

[mgg31240-bib-0025] Iskender, C. , Kartal, E. , Akcimen, F. , Kocoglu, C. , Ozoguz, A. , Kotan, D. , … Basak, A. N. (2015). Turkish families with juvenile motor neuron disease broaden the phenotypic spectrum of SPG11. Neurology Genetics, 1(3), e25 10.1212/NXG.0000000000000025 27066562 PMC4809458

[mgg31240-bib-0026] Kara, E. , Tucci, A. , Manzoni, C. , Lynch, D. S. , Elpidorou, M. , Bettencourt, C. , … Houlden, H. (2016). Genetic and phenotypic characterization of complex hereditary spastic paraplegia. Brain, 139(Pt 7), 1904–1918. 10.1093/brain/aww111 27217339 PMC4939695

[mgg31240-bib-0027] Khani, M. , Alavi, A. , Shamshiri, H. , Zamani, B. , Hassanpour, H. , Kazemi, M. H. , Elahi, E. (2019). Mutation screening of SLC52A3, C19orf12, and TARDBP in Iranian ALS patients. Neurobiology of Aging, 75, 225.e9–225.e14. 10.1016/j.neurobiolaging.2018.11.003 30553531

[mgg31240-bib-0028] Lee, M. J. , Cheng, T. W. , Hua, M. S. , Pan, M. K. , Wang, J. , Stephenson, D. A. , & Yang, C. C. (2008). Mutations of the SPG11 gene in patients with autosomal recessive spastic paraparesis and thin corpus callosum. Journal of Neurology, Neurosurgery and Psychiatry, 79(5), 607–609. 10.1136/jnnp.2007.136390 18408091

[mgg31240-bib-0029] Lupski, J. R. , Reid, J. G. , Gonzaga‐Jauregui, C. , Rio Deiros, D. , Chen, D. C. Y. , Nazareth, L. , … Gibbs, R. A. (2010). Whole‐genome sequencing in a patient with Charcot‐Marie‐Tooth neuropathy. New England Journal of Medicine, 362(13), 1181–1191. 10.1056/NEJMoa0908094 20220177 PMC4036802

[mgg31240-bib-0030] Marttila, M. , Kytövuori, L. , Helisalmi, S. , Kallio, M. , Laitinen, M. , Hiltunen, M. , … Majamaa, K. (2017). Molecular epidemiology of Charcot‐Marie‐Tooth disease in Northern Ostrobothnia, Finland: A population‐based study. Neuroepidemiology, 49(1–2), 34–39. 10.1159/000478860 28810241

[mgg31240-bib-0031] Mathis, S. , Goizet, C. , Tazir, M. , Magdelaine, C. , Lia, A. S. , Magy, L. , & Vallat, J. M. (2015). Charcot‐Marie‐Tooth diseases: An update and some new proposals for the classification. Journal of Medical Genetics, 52(10), 681–690. 10.1136/jmedgenet-2015-103272 26246519

[mgg31240-bib-0032] Meyer, T. , Schwan, A. , Dullinger, J. S. , Brocke, J. , Hoffmann, K.‐T. , Nolte, C. H. , … Linke, P. (2005). Early‐onset ALS with long‐term survival associated with spastin gene mutation. Neurology, 65(1), 141–143. 10.1212/01.wnl.0000167130.31618.0a 16009903

[mgg31240-bib-0033] Montecchiani, C. , Pedace, L. , Lo Giudice, T. , Casella, A. , Mearini, M. , Gaudiello, F. , … Orlacchio, A. (2016). ALS5/SPG11/KIAA1840 mutations cause autosomal recessive axonal Charcot‐Marie‐Tooth disease. Brain, 139(Pt 1), 73–85. 10.1093/brain/awv320 26556829 PMC5839554

[mgg31240-bib-0034] Mukai, M. , Koh, K. , Ohnuki, Y. , Nagata, E. , Takiyama, Y. , & Takizawa, S. (2018). Novel SPG11 mutations in a patient with symptoms mimicking multiple sclerosis. Internal Medicine, 57(21), 3183–3186. 10.2169/internalmedicine.0976-18 29877287 PMC6262711

[mgg31240-bib-0035] Nelson, L. M. (1995). Epidemiology of ALS. Clinical Neuroscience, 3(6), 327–331. 9021253

[mgg31240-bib-0036] Novarino, G. , Fenstermaker, A. G. , Zaki, M. S. , Hofree, M. , Silhavy, J. L. , Heiberg, A. D. , … Gleeson, J. G. (2014). Exome sequencing links corticospinal motor neuron disease to common neurodegenerative disorders. Science, 343(6170), 506–511. 10.1126/science.1247363 24482476 PMC4157572

[mgg31240-bib-0037] Orlacchio, A. , Babalini, C. , Borreca, A. , Patrono, C. , Massa, R. , Basaran, S. , … Kawarai, T. (2010). SPATACSIN mutations cause autosomal recessive juvenile amyotrophic lateral sclerosis. Brain, 133(Pt 2), 591–598. 10.1093/brain/awp325 20110243 PMC2822627

[mgg31240-bib-0038] Orlen, H. , Melberg, A. , Raininko, R. , Kumlien, E. , Entesarian, M. , Soderberg, P. , … Dahl, N. (2009). SPG11 mutations cause Kjellin syndrome, a hereditary spastic paraplegia with thin corpus callosum and central retinal degeneration. American Journal of Medical Genetics. Part B, Neuropsychiatric Genetics: The Official Publication of the International Society of Psychiatric Genetics., 150B(7), 984–992. 10.1002/ajmg.b.30928 19194956

[mgg31240-bib-0039] Paisan‐Ruiz, C. , Dogu, O. , Yilmaz, A. , Houlden, H. , & Singleton, A. (2008). SPG11 mutations are common in familial cases of complicated hereditary spastic paraplegia. Neurology, 70(16 Pt 2), 1384–1389. 10.1212/01.wnl.0000294327.66106.3d 18337587 PMC2730021

[mgg31240-bib-0040] Pérez‐Brangulí, F. , Mishra, H. K. , Prots, I. , Havlicek, S. , Kohl, Z. , Saul, D. , … Winner, B. (2014). Dysfunction of spatacsin leads to axonal pathology in SPG11‐linked hereditary spastic paraplegia. Human Molecular Genetics, 23(18), 4859–4874. 10.1093/hmg/ddu200 24794856 PMC4140466

[mgg31240-bib-0041] Querin, G. , Bertolin, C. , Martinuzzi, A. , Bassi, M. T. , Arnoldi, A. , Polo, A. , … Soraru, G. (2014). The blurred scenario of motor neuron disorders linked to Spatacsin mutations: A case report. European Journal of Neurology, 21(10), e85–86. 10.1111/ene.12481 25209065

[mgg31240-bib-0042] Ruano, L. , Melo, C. , Silva, M. C. , & Coutinho, P. (2014). The global epidemiology of hereditary ataxia and spastic paraplegia: A systematic review of prevalence studies. Neuroepidemiology, 42(3), 174–183. 10.1159/000358801 24603320

[mgg31240-bib-0043] Sawada, H. (2017). Clinical efficacy of edaravone for the treatment of amyotrophic lateral sclerosis. Expert Opinion on Pharmacotherapy, 18(7), 735–738. 10.1080/14656566.2017.1319937 28406335

[mgg31240-bib-0044] Sawalha, K. , Gonzalez‐Toledo, E. , & Hussein, O. (2019). Role of magnetic resonance imaging in diagnosis of motor neuron disease: literature review and two case illustrations. The Permanente Journal, 23, 10.7812/TPP/18-131 PMC6380476 30939271

[mgg31240-bib-0045] Schneider‐Gold, C. , Dekomien, G. , Regensburger, M. , Schneider, R. , Trampe, N. , Krogias, C. , … Bellenberg, B. (2017). Monozygotic twins with a new compound heterozygous SPG11 mutation and different disease expression. Journal of the Neurological Sciences, 381, 265–268. 10.1016/j.jns.2017.09.005 28991695

[mgg31240-bib-0046] Schule, R. , & Schols, L. (2011). Genetics of hereditary spastic paraplegias. Seminars in Neurology, 31(5), 484–493. 10.1055/s-0031-1299787 22266886

[mgg31240-bib-0047] Schüle, R. , Wiethoff, S. , Martus, P. , Karle, K. N. , Otto, S. , Klebe, S. , … Schöls, L. (2016). Hereditary spastic paraplegia: Clinicogenetic lessons from 608 patients. Annals of Neurology, 79(4), 646–658. 10.1002/ana.24611 26856398

[mgg31240-bib-0048] Stevanin, G. , Azzedine, H. , Denora, P. , Boukhris, A. , Tazir, M. , Lossos, A. , … Durr, A. (2008). Mutations in SPG11 are frequent in autosomal recessive spastic paraplegia with thin corpus callosum, cognitive decline and lower motor neuron degeneration. Brain, 131(Pt 3), 772–784. 10.1093/brain/awm293 18079167

[mgg31240-bib-0049] Stevanin, G. , Santorelli, F. M. , Azzedine, H. , Coutinho, P. , Chomilier, J. , Denora, P. S. , … Brice, A. (2007). Mutations in SPG11, encoding spatacsin, are a major cause of spastic paraplegia with thin corpus callosum. Nature Genetics, 39(3), 366–372. 10.1038/ng1980 17322883

[mgg31240-bib-0050] Strong, M. J. , & Gordon, P. H. (2005). Primary lateral sclerosis, hereditary spastic paraplegia and amyotrophic lateral sclerosis: Discrete entities or spectrum? Amyotrophic Lateral Sclerosis Other Motor Neuron Disorders, 6(1), 8–16. 10.1080/14660820410021267 16036421

[mgg31240-bib-0051] Wakil, S. M. , Ramzan, K. , Abuthuraya, R. , Hagos, S. , Al‐Dossari, H. , Al‐Omar, R. , … Bohlega, S. (2014). Infantile‐onset ascending hereditary spastic paraplegia with bulbar involvement due to the novel ALS2 mutation c.2761C>T. Gene, 536(1), 217–220. 10.1016/j.gene.2013.11.043 24315819

[mgg31240-bib-0052] Wijesekera, L. C. , & Leigh, P. N. (2009). Amyotrophic lateral sclerosis. Orphanet Journal of Rare Diseases, 4, 3 10.1186/1750-1172-4-3 19192301 PMC2656493

[mgg31240-bib-0053] Yang, Y. I. , Hentati, A. , Deng, H.‐X. , Dabbagh, O. , Sasaki, T. , Hirano, M. , … Siddique, T. (2001). The gene encoding alsin, a protein with three guanine‐nucleotide exchange factor domains, is mutated in a form of recessive amyotrophic lateral sclerosis. Nature Genetics, 29(2), 160–165. 10.1038/ng1001-160 11586297

[mgg31240-bib-0054] Yildirim, Y. , Orhan, E. K. , Iseri, S. A. , Serdaroglu‐Oflazer, P. , Kara, B. , Solakoglu, S. , & Tolun, A. (2011). A frameshift mutation of ERLIN2 in recessive intellectual disability, motor dysfunction and multiple joint contractures. Human Molecular Genetics, 20(10), 1886–1892. 10.1093/hmg/ddr070 21330303

[mgg31240-bib-0055] Zhang, S. S. , Chen, Q. , Chen, X. P. , Wang, J. G. , Burgunder, J. M. , Shang, H. F. , & Yang, Y. (2008). Two novel mutations in the SPG11 gene causing hereditary spastic paraplegia associated with thin corpus callosum. Movement Disorders, 23(6), 917–919. 10.1002/mds.21942 18361476

